# Low Vitamin D Status and Its Association With Systemic Inflammation and Immune Dysregulation: A Retrospective Cross-Sectional Study

**DOI:** 10.7759/cureus.108165

**Published:** 2026-05-03

**Authors:** Ahmad Al-Ajaj, Wissem Melki, Tarek Mansour Abugalala

**Affiliations:** 1 Internal Medicine, Al-Ahli Hospital, Doha, QAT

**Keywords:** 25-hydroxyvitamin d, cross-sectional analysis, eosinophilia, erythrocyte sedimentation rate, immune dysregulation, immune response, inflammatory markers, retrospective cross-sectional study, systemic inflammation, vitamin d deficiency

## Abstract

Background and objective

Vitamin D contributes to immune regulation and the control of inflammatory signaling. Deficient vitamin D status has been linked to greater systemic inflammatory activity, but its relationship with routinely used clinical markers such as erythrocyte sedimentation rate (ESR) and absolute eosinophil count (AEC) remains insufficiently characterized. The objective of this study is to assess whether low vitamin D status is associated with elevated ESR and eosinophilia in an internal medicine outpatient population.

Methods

This retrospective cross-sectional study included 200 patients evaluated at the Internal Medicine Clinic of Al-Ahli Hospital, Doha, Qatar, between January 1, 2022, and April 1, 2026. Participants were assigned to two equal groups according to serum 25-hydroxyvitamin D level: low vitamin D (n = 100) and normal (n = 100). For the purposes of this study, low vitamin D status was defined as serum 25-hydroxyvitamin D <30 ng/mL, combining deficiency and insufficiency categories, while normal vitamin D status was defined as 30-100 ng/mL. Elevated ESR was determined using age- and sex-specific thresholds, and eosinophilia was defined as an AEC >0.5 × 10⁹/L. Categorical comparisons were performed with Fisher’s exact test.

Results

Vitamin D deficiency was associated with a higher prevalence of elevated ESR, identified in 23% of the low vitamin D group compared with 10% of the normal vitamin D group (relative risk (RR) 2.3; p = 0.021). Eosinophilia was also more frequent in the low vitamin D group, observed in 14% of patients versus 4% in those with normal vitamin D status (RR 3.5; p < 0.05). These findings demonstrate a significant association between low vitamin D levels and increased systemic inflammatory activity. Additionally, the higher prevalence of eosinophilia suggests a potential link between vitamin D deficiency and immune dysregulation.

Conclusions

Low vitamin D status was significantly associated with elevated ESR, supporting an association with systemic inflammatory activity. Eosinophilia was more frequent in the low vitamin D group, and this difference was statistically significant. Overall, these findings suggest that low vitamin D status may accompany broader inflammatory and immunologic imbalance.

## Introduction

Low vitamin D status is common worldwide and is increasingly recognized as a problem that extends beyond bone health. In addition to its established role in calcium and phosphate homeostasis, vitamin D influences immune regulation, inflammatory signaling, and host defense mechanisms [1,2]. Serum 25-hydroxyvitamin D is the accepted marker of vitamin D status, and low levels remain prevalent in many populations, including those living in sun-rich environments [1,2].

Vitamin D receptors and vitamin D-metabolizing enzymes are present in monocytes, macrophages, dendritic cells, and activated lymphocytes. Through these pathways, vitamin D can modify innate and adaptive immune responses, influence cytokine expression, and help preserve immunologic balance [3-5]. When vitamin D status is low, this regulatory effect may be reduced, creating conditions that favor a more pro-inflammatory profile [3-5].

This issue is especially relevant in Qatar and the broader Gulf region. Despite year-round sunlight, studies from Qatar have repeatedly shown a high prevalence of vitamin D deficiency or insufficiency in routine clinical populations [6-8]. Proposed contributors include limited sun exposure, indoor lifestyles, cultural clothing practices, and differences in skin pigmentation [6-8]. Because of this high background prevalence, understanding the clinical correlates of low vitamin D status in Qatar remains important.

Among the most widely used inflammatory tests in daily practice is the erythrocyte sedimentation rate (ESR), an inexpensive marker that can reflect systemic inflammatory activity. Prior studies examining the relationship between low vitamin D status and inflammatory indices have produced mixed but suggestive findings. Some reports have described inverse associations between vitamin D levels and ESR or related hematologic inflammatory markers, whereas others have found weaker or more context-dependent effects [9-11].

Eosinophils are another potentially relevant marker because they participate in allergic, parasitic, and immune-mediated inflammation. Several studies, particularly in asthma and other allergic conditions, have linked lower vitamin D levels to higher eosinophil counts, higher IgE levels, poorer disease control, or greater exacerbation risk [12-20]. However, most of that data comes from selected respiratory or pediatric cohorts rather than heterogeneous outpatient internal medicine populations.

Accordingly, this study evaluated the association between low vitamin D status and two accessible laboratory markers, ESR and absolute eosinophil count (AEC), in a diverse outpatient population attending an internal medicine clinic in Doha, Qatar. By studying both Qatari and non-Qatari patients with varied backgrounds, occupations, activity patterns, and skin pigmentation, this study provides additional clinical evidence about the inflammatory correlates of low vitamin D status in routine clinical care [6-8,21,22].

## Materials and methods

Study design and setting

This work used a retrospective cross-sectional design. It was conducted at the Internal Medicine Clinic of Al-Ahli Hospital in Doha, Qatar, and covered the period from January 1, 2022, through April 1, 2026. The objective was to determine whether vitamin D status was associated with selected laboratory markers of systemic inflammation and immune dysregulation.

Ethical approval

The study protocol was reviewed and approved by the Institutional Ethics Committee of Al-Ahli Hospital, Doha, Qatar (approval EC2026/02). The study followed the principles of the Declaration of Helsinki. Because the analysis relied on previously recorded, anonymized medical record data, the requirement for informed consent was waived.

Study population and group allocation

A total of 200 patients met the study criteria. Patients were classified into two equal groups according to serum 25-hydroxyvitamin D level: a low vitamin D group (n = 100) and a normal vitamin D group (n = 100). Group allocation was based on the most recent qualifying laboratory results available during the study period. The study population reflected the diversity of the clinic’s catchment area and included both Qatari and non-Qatari individuals from multiple world regions, with varied occupations, activity patterns, and skin pigmentation.

Inclusion and exclusion criteria

Patients were eligible if they were 14 years of age or older, of either sex, and had complete laboratory data from the same clinical encounter. Required data elements were serum vitamin D level, complete blood count, and ESR. No additional exclusion criteria were applied beyond missing or incomplete required data.

Data sources and variables

Data were retrieved from the Al-Ahli Hospital electronic medical record system. The variables collected included age, sex, BMI, serum 25-hydroxyvitamin D level, complete blood count parameters, including AEC, and ESR. Only laboratory measurements obtained at the same visit were analyzed in order to reduce temporal variation between tests.

Laboratory measurements and operational definitions

Serum 25-hydroxyvitamin D was measured by chemiluminescence immunoassay. For the purposes of this study, low vitamin D status was defined as serum 25-hydroxyvitamin D <30 ng/mL, combining deficiency and insufficiency categories, while normal vitamin D status was defined as 30-100 ng/mL. ESR was measured by the Westergren method. Complete blood count testing was performed on the Siemens ADVIA 2120i automated hematology system (Siemens Healthineers, Erlangen, Germany). Elevated ESR was defined using age- and sex-specific thresholds: men <50 years >15 mm/hr, men ≥50 years >20 mm/hr, women <50 years >20 mm/hr, and women ≥50 years >30 mm/hr. Eosinophilia was defined as an AEC >0.5 × 10⁹/L.

Statistical analysis

Categorical variables were compared between groups using Fisher’s exact test. Relative risk was calculated to estimate the strength of association between low vitamin D status and each inflammatory outcome. A p-value <0.05 was considered statistically significant. Analyses were performed in Microsoft Excel (Microsoft Corporation, Redmond, WA, USA).

Confidentiality

All data were anonymized before analysis, and no patient-identifying information was included in the study dataset or manuscript.

## Results

Cohort overview

The final analytic sample comprised 200 patients, with 100 patients in the low vitamin D group and 100 patients in the normal vitamin D group. The cohort included male and female participants, adolescents and adults aged 14 years and older, and both Qatari and non-Qatari individuals drawn from a heterogeneous outpatient population.

Because the study was designed around the availability of simultaneous vitamin D, ESR, and complete blood count measurements, all 200 included patients had complete outcome data for the primary comparisons. Table 1 summarizes the available baseline descriptors of the study population.

**Table 1:** Baseline characteristics of the study population Values are presented as mean ± SD where applicable.

Characteristic	Low vitamin D (n = 100)	Normal vitamin D (n = 100)
Age (years, mean ± SD)	37.2 ± 11.0	41.3 ± 12.5
Sex (male/female)	59/41	50/50
BMI (kg/m², mean ± SD)	27.2 ± 4.9	27.0 ± 4.0
Mean vitamin D (ng/mL)	17.7 ± 6.1	41.6 ± 11.5
Nationality	Qatari and non-Qatari	Qatari and non-Qatari
Geographic background	Multiple regions	Multiple regions
Occupation/activity	Diverse	Diverse
Skin pigmentation	Broad spectrum	Broad spectrum

Primary outcome analysis

A higher proportion of patients in the low vitamin D group had an elevated ESR than patients with normal vitamin D status. Specifically, 23 of 100 patients in the low vitamin D group met the elevated ESR definition, compared with 10 of 100 patients in the normal vitamin D group. This corresponded to a relative risk of 2.3 and a p-value of 0.021, indicating a statistically significant association between low vitamin D status and elevated ESR.

Eosinophilia was also more frequent in the low vitamin D group. Fourteen of 100 patients in the low vitamin D group had an AEC above 0.5 × 10⁹/L, compared with four of 100 patients in the normal vitamin D group. The relative risk was 3.5, and this difference reached statistical significance (p < 0.05). Table 2 presents the comparative outcome analysis for the two study groups.

**Table 2:** Comparative outcome analysis for the two study groups Data are presented as n (%). Group comparisons were performed using Fisher’s exact test. Relative risk and p-values are shown in the table.

Outcome	Low vitamin D, n (100 patients)	Normal vitamin D, n (100 patients)	Relative risk	p-Value
Elevated ESR	23 (23%)	10 (10%)	2.3	0.021
AEC >0.5 × 10⁹/L	14 (14%)	4 (4%)	3.5	p < 0.05

Summary of findings

The results demonstrate a clearer relationship between low vitamin D status and elevated ESR than between low vitamin D status and eosinophilia. The ESR finding reached statistical significance and suggests that low vitamin D status may accompany higher systemic inflammatory activity in this outpatient population.

The eosinophil result showed the same direction of effect, with a higher event rate in the low vitamin D group and an elevated relative risk. Although this comparison was statistically significant, the magnitude of the observed difference supports the possibility of an underlying biologic relationship that would merit evaluation in a larger sample.

## Discussion

In this retrospective cross-sectional analysis, low vitamin D status was associated with a significantly greater frequency of elevated ESR and a higher frequency of eosinophilia. These findings support the concept that reduced vitamin D levels may coexist with measurable systemic inflammatory and immunologic alterations in routine outpatient internal medicine practice.

The association between low vitamin D status and elevated ESR is biologically plausible. Vitamin D plays a well-established role in modulating both innate and adaptive immune responses. It influences macrophage activity, dendritic cell maturation, and T-lymphocyte differentiation, while also regulating cytokine production, including IL-6, tumor necrosis factor-alpha, and other mediators of inflammation [3-5]. When vitamin D levels are reduced, these immunoregulatory effects may be diminished, leading to a shift toward a more pro-inflammatory state. ESR, although nonspecific, reflects the presence of systemic inflammatory proteins such as fibrinogen and immunoglobulins and therefore serves as a practical surrogate marker of inflammatory activity. The higher prevalence of elevated ESR observed in the low vitamin D group is therefore consistent with this biologic framework.

The present findings are in agreement with prior clinical studies. Yildirim et al. demonstrated higher ESR and other inflammatory markers in vitamin D-deficient patients, both in the presence and absence of chronic kidney disease [9]. Similarly, Kaya et al. reported increased ESR values in patients with type 2 diabetes and low vitamin D levels, with an inverse correlation between vitamin D concentration and ESR [10]. Wang et al. further identified an association between vitamin D status and the neutrophil-to-lymphocyte ratio, suggesting a broader relationship between vitamin D and hematologic indices of inflammation [11]. Although these studies were conducted in more selected populations, the consistency in direction of effect supports the generalizability of the association observed in the current heterogeneous outpatient cohort.

The eosinophil findings also warrant careful consideration. Eosinophilia was significantly more frequent in the low vitamin D group, with a relative risk greater than 1, indicating a potential association between vitamin D status and eosinophil-mediated inflammatory pathways. Eosinophils are key effector cells in allergic, parasitic, and certain immune-mediated conditions, and their activity is closely linked to T-helper 2 (Th2)-mediated immune responses. Vitamin D has been shown to influence T-cell polarization and cytokine expression, including IL-4, IL-5, and IL-13, which are central to eosinophil activation and survival [3-5,16].

These findings are supported by previous studies. Souto Filho et al. reported an association between vitamin D deficiency and increased peripheral eosinophil counts [12]. In pediatric asthma populations, de Amorim et al. demonstrated that lower vitamin D levels were associated with higher eosinophil counts and elevated IgE levels [13]. Additional studies by Dogru et al. and Brehm et al. have shown links between low vitamin D status and increased asthma severity, exacerbation risk, and markers of allergic inflammation [14,15]. While many of these studies were conducted in selected respiratory cohorts, the present study extends these observations to a broader internal medicine population, suggesting that the relationship between vitamin D and eosinophil-associated inflammation may not be limited to specific disease groups.

From a clinical perspective, the use of ESR and AEC as markers in this study is particularly relevant. Both tests are inexpensive, widely available, and routinely performed in outpatient settings. The observed associations suggest that low vitamin D status may serve as a useful contextual marker when interpreting these laboratory parameters. Although vitamin D measurement is not currently part of standard inflammatory assessment, its relationship with commonly used markers such as ESR and eosinophils may provide additional insight into underlying inflammatory or immunologic processes in everyday clinical practice.

This study has several strengths. It was conducted in Qatar, a region characterized by a high prevalence of low vitamin D status despite abundant sunlight [6-8,21]. The study population was heterogeneous and reflective of routine clinical practice, including both Qatari and non-Qatari patients with diverse backgrounds, occupations, and levels of sun exposure. Furthermore, all included patients had simultaneous measurements of vitamin D, ESR, and complete blood count parameters, minimizing temporal variability between exposure and outcome assessment.

However, several limitations should be acknowledged. The retrospective cross-sectional design limits the ability to establish causality and does not allow the determination of temporal relationships between vitamin D status and inflammatory markers. Residual confounding is possible, as ESR can be influenced by factors such as infection, anemia, and chronic disease, while eosinophil counts may be affected by allergic conditions, parasitic infections, asthma, and medication use. In addition, the study did not include multivariable adjustment for potential confounders, and the sample size, although adequate for detecting ESR differences, may still limit the precision of estimates for eosinophil-related outcomes. Future studies with larger cohorts and prospective designs would be valuable to further clarify these associations and explore potential mechanisms.

Overall, the present study contributes to the growing body of evidence linking low vitamin D status with markers of systemic inflammation and immune dysregulation. The findings demonstrate that low vitamin D status in a heterogeneous internal medicine outpatient population is associated with a significant increase in elevated ESR and a significant increase in eosinophilia. These observations are consistent with a broader role of vitamin D in modulating inflammatory and immune pathways and support further investigation into its clinical implications.

## Conclusions

Within this outpatient cohort from Doha, Qatar, low vitamin D status was significantly associated with elevated ESR and a higher frequency of eosinophilia. The association with elevated ESR supports a link between low vitamin D and increased systemic inflammatory activity, while the higher prevalence of eosinophilia suggests a potential relationship with immune or allergic dysregulation. These findings underscore the possible clinical relevance of vitamin D status in interpreting commonly used laboratory markers in routine outpatient practice, particularly given the high prevalence of vitamin D deficiency and its potential implications for patient assessment and risk stratification. However, due to the retrospective cross-sectional design, causality cannot be established. Further prospective and mechanistic studies are needed to clarify the nature of these associations and to determine whether correction of low vitamin D status can favorably influence inflammatory and immunologic outcomes.

## References

[1] Holick MF (2007). Vitamin D deficiency. N Engl J Med.

[2] Holick MF, Binkley NC, Bischoff-Ferrari HA (2011). Evaluation, treatment, and prevention of vitamin D deficiency: an Endocrine Society clinical practice guideline. J Clin Endocrinol Metab.

[3] Charoenngam N, Holick MF (2020). Immunologic effects of vitamin D on human health and disease. Nutrients.

[4] Sîrbe C, Rednic S, Grama A, Pop TL (2022). An update on the effects of vitamin D on the immune system and autoimmune diseases. Int J Mol Sci.

[5] Fletcher J, Bishop EL, Harrison SR (2022). Autoimmune disease and interconnections with vitamin D. Endocr Connect.

[6] Badawi A, Arora P, Sadoun E, Al-Thani AA, Thani MH (2012). Prevalence of vitamin D insufficiency in Qatar: a systematic review. J Public Health Res.

[7] Zainel AA, Qotba H, Al Nuaimi A, Syed M (2019). Vitamin D status among adults (18-65 years old) attending primary healthcare centres in Qatar: a cross-sectional analysis of the electronic medical records for the year 2017. BMJ Open.

[8] Al-Dabhani K, Tsilidis KK, Murphy N (2017). Prevalence of vitamin D deficiency and association with metabolic syndrome in a Qatari population. Nutr Diabetes.

[9] Yildirim I, Hur E, Kokturk F (2013). Inflammatory markers: C-reactive protein, erythrocyte sedimentation rate, and leukocyte count in vitamin D deficient patients with and without chronic kidney disease. Int J Endocrinol.

[10] Kaya T, Akçay EÜ, Ertürk Z, Ergenç H, Tamer A (2018). The relationship between vitamin D deficiency and erythrocyte sedimentation rate in patients with diabetes. Turk J Med Sci.

[11] Wang SY, Shen TT, Xi BL, Shen Z, Zhang X (2021). Vitamin D affects the neutrophil-to-lymphocyte ratio in patients with type 2 diabetes mellitus. J Diabetes Investig.

[12] Souto Filho JT, de Andrade AS, Ribeiro FM, Alves PA, Simonini VR (2018). Impact of vitamin D deficiency on increased blood eosinophil counts. Hematol Oncol Stem Cell Ther.

[13] de Amorim CL, Oliveira JM, Rodrigues A, Furlanetto KC, Pitta F (2020). Vitamin D: association with eosinophil counts and IgE levels in children with asthma. J Bras Pneumol.

[14] Dogru M, Kirmizibekmez H, Yesiltepe Mutlu RG, Aktas A, Ozturkmen S (2014). Clinical effects of vitamin D in children with asthma. Int Arch Allergy Immunol.

[15] Brehm JM, Celedón JC, Soto-Quiros ME (2009). Serum vitamin D levels and markers of severity of childhood asthma in Costa Rica. Am J Respir Crit Care Med.

[16] Pfeffer PE, Mann EH, Hornsby E, Chambers ES, Chen YH, Rice L, Hawrylowicz CM (2014). Vitamin D influences asthmatic pathology through its action on diverse immunological pathways. Ann Am Thorac Soc.

[17] Majak P, Olszowiec-Chlebna M, Smejda K, Stelmach I (2011). Vitamin D supplementation in children may prevent asthma exacerbation triggered by acute respiratory infection. J Allergy Clin Immunol.

[18] Fares MM, Alkhaled LH, Mroueh SM, Akl EA (2015). Vitamin D supplementation in children with asthma: a systematic review and meta-analysis. BMC Res Notes.

[19] Wang Q, Ying Q, Zhu W, Chen J (2022). Vitamin D and asthma occurrence in children: a systematic review and meta-analysis. J Pediatr Nurs.

[20] El Abd A, Dasari H, Dodin P, Trottier H, Ducharme FM (2024). Associations between vitamin D status and biomarkers linked with inflammation in patients with asthma: a systematic review and meta-analysis of interventional and observational studies. Respir Res.

[21] Ganji V, Sukik A, Alaayesh H, Rasoulinejad H, Shraim M (2020). Serum vitamin D concentrations are inversely related to prevalence of metabolic syndrome in Qatari women. Biofactors.

[22] Bouillon R, Manousaki D, Rosen C, Trajanoska K, Rivadeneira F, Richards JB (2022). The health effects of vitamin D supplementation: evidence from human studies. Nat Rev Endocrinol.

